# Comparison of dural grafts in Chiari decompression surgery: Review of the literature

**DOI:** 10.4103/0974-8237.65479

**Published:** 2010

**Authors:** Adib A. Abla, Timothy Link, David Fusco, David A. Wilson, Volker K.H. Sonntag

**Affiliations:** Division of Neurological Surgery, Barrow Neurological Institute, St. Joseph's Hospital and Medical Center, Phoenix, Arizona

**Keywords:** Chiari malformation, duraplasty, pericranium

## Abstract

**Background:**

Decompression of Chiari malformation is a common procedure in both pediatric and adult neurosurgery. Although the necessity for some bony removal is universally accepted, other aspects of Chiari surgery are the subject of debate. The most controversial points include the optimal amount of bony removal, the use of duraplasty (and the type of material), the need for subarachnoid dissection, and the need for tonsillar shrinkage.

**Material and Methods:**

We critically reviewed the literature to elucidate the risks and benefits of different graft types and to clarify optimal treatment options therein. Based on our search results, 108 relevant articles were identified. With specific inclusion and exclusion criteria, we noted three studies that directly compared two tlpes of dural substitutes in Chiari malformation surgery.

**Results:**

Our review did not support the superiority of either autologous or nonautologous grafts when duraplasty is employed. Our institutional experience, however, dictates that when the pericranium is available and of good quality, it should be utilized for duraplasty. It is non-immunogenic, inexpensive, and capable of creating a watertight closure with the dura.

**Conclusions:**

Discrepancies between the three comparative studies analyzed are likely attributable to increases in pericranial quality and thickness with maturity. Future randomized studies with large numbers and the power to resolve differences in the relatively low rates of complications in Chiari surgery are warranted.

## INTRODUCTION

Decompression of Chiari malformations is commonly performed in both pediatric and adult neurosurgery. The surgery involves a suboccipital craniectomy and, in most cases, a C1 laminectomy, to relieve the spectrum of symptoms associated with tonsillar herniation below the foramen magnum.[1] Chiari decompression is also an effective treatment for syringomyelia, which is frequently associated with Chiari malformations.[2] The proposed pathological mechanism underlying the progressive symptoms of Chiari malformation involves tonsillar obstruction of the normal flow of cerebrospinal fluid (CSF) between the spinal and cortical subarachnoid spaces. This disturbance can lead to syringomyelia, CSF trapped in the spinal subarachnoid space, spinal cord compression, and increased intracranial pressure.[3] By expanding a crowded foramen magnum, Chiari decompression both relieves cervicomedullary compression and restores physiologic dynamics of CSF flow.

Although decompression of Chiari malformation is a proven and effective treatment, several variations of the procedure have been proposed. However, there is no clear consensus among the neurosurgical community regarding the most efficacious technique. Although the necessity for some bony removal is universally accepted, other aspects of Chiari surgery are debated. The most controversial points include the optimal amount of bony removal, the need for duraplasty (and the type of material), the need for subarachnoid dissection, and the need for tonsillar shrinkage.[2,4‐9] Additional variables include the use of postclosure dural/graft sealants, the type of dural/graft suture, whether to tent the dural graft to the remaining bone or a suboccipital metallic plate, and the use of adjunctive syrinx shunting when a syrinx is present.

The proponents of bony decompression alone assert that this method affords protection from postoperative complications related to dural opening and entering the subarachnoid space. Although the optimal amount of bony removal remains undefined,[2] bony decompression without duraplasty decreases operative time and hospital stays.[5] Furthermore, brainstem auditory-evoked potentials improved the most after bony decompression with little additional gain associated with dural opening.[4,9] Some authors have advocated the use of intraoperative ultrasonography as a means for assessing tonsillar compression after bony removal, with persistent compression or lack of pulsation supporting the need for dural opening and duraplasty.[6‐8]

Despite the lack of consensus on which surgical methods are necessary and sufficient to relieve Chiari pathology, most neurosurgeons perform both bony decompression and duraplasty.[2] In a recent meta-analysis that reviewed seven series with 582 patients altogether, posterior fossa decompression with duraplasty was associated with a lower risk of reoperation than bony decompression alone but with a greater risk of CSF-related complications. The reviewers did not stratify the data by type of graft. Interestingly, they found no statistically significant difference between the treatments in terms of clinical improvement or resolution of syrinx.[3]

Although duraplasty remains popular and has many advocates,[1,10,11] the optimal type of dural graft is debated. A number of graft types, both autologous and nonautologous, are available to the neurosurgeon. Dural substitutes used in Chiari I decompression surgery include autologous pericranium,[1,10,12‐14] bovine pericardium,[12] cadaveric dura,[1,12] synthetic bovine collagen matrix (Duragen, Integra Neuroscience, Plainsboro, NJ),[11] acellular human dermis allograft (AlloDerm, LifeCell Corp., Branchburg, NJ),[11,15] autologous fascia lata,[12,16] expanded polytetrafluoroethylene (PTFE),[10,17,18] posterior atlantooccipital membrane,[19] splenius capitis muscle flap,[20] and porcine small intestinal submucosa (Durasis, Cook Biotech, Inc., West Lafayette, IN).[21] A recent survey of pediatric neurosurgeons by the American Association of Neurological Surgeons estimated graft preferences: 32% preferred autologous pericranium, 32% preferred bovine pericardium, 17% preferred lyophilized cadaveric dura, 16% preferred synthetic products, 4% preferred ligamentum nuchae, and 3% preferred fascia lata.[22] When a dural opening is made for a Chiari I decompression, these grafts are used to create room for the cerebellar tonsils by expanding the potential space posterior to the hindbrain at the foramen magnum. Theoretically, the ideal graft would provide watertight closure without promoting arachnoid scarring or provoking an inflammatory response.

The current literature reflects the debate regarding the impact of dural graft material on several clinical outcomes measures, most notable are the need for reoperation and the incidences of post-operative CSF leak, pseudomeningocele fromation, meningitis (bacterial or aseptic), wound breakdown. Adverse reactions to nonautologous (e.g., synthetic, allogenic, xenogenic) grafts include graft dissolution, encapsulation, foreign-body reaction, excessive scarring, and adhesion formation.[21] After initially successful Chiari I surgery, many recurrences may be due to a foreign body reaction, scarring, intradural adhesions, and meningitis-induced hydrocephalus.[4,23,24] The thickness and assimilation of the graft can also affect its ease of manipulation, workability, and role as a watertight sealant to prevent extradural egress of CSF and ingress of blood and contaminants.[14] We performed a critical review of the literature to elucidate the risks and benefits of different graft types and to clarify optimal treatment options.

## MATERIAL AND METHODS

### Search criteria

The search terms "Chiari Malformation AND Duraplasty," "Chiari Malformation AND Pericranium," "Chiari AND Graft AND Duraplasty," and "Chiari Malformation AND Graft" were separately entered in a Medline search in PubMed (November 7, 2009).

### Study selection

The inclusion criteria were as follows: articles that involved Chiari I/II malformation surgery, articles that addressed 'duraplasty' in the title, and articles that evaluated two separate dural substitutes.

The exclusion criteria were as follows: articles that did not specifically report rates of resolution of reoperation or complications including meningitis, CSF leaks, and wound infections; articles comparing outcomes obtained with bony decompression alone versus those obtained with bony decompression and duraplasty; articles that reported outcomes in fewer than 10 patients; and articles written in a language other than English.

### Statistical analysis

Data analysis was performed using the Fisher-exact test and Chi-square test for independence of categorical variables. All probability values were two-sided, and a *P*-value less than 0.05 was considered to be statistically significant.

## RESULTS

### Search results

Based on the search results, 108 articles were identified. After applying the inclusion and exclusion criteria, we identified three studies that compared two types of dural substitutes in Chiari malformation surgery. A fourth study[25] comparing pericranium versus synthetic graft was identified.[14] However, it dealt with brain tumor resection in addition to Chiari decompression and was therefore excluded. A fifth study comparing autologous and nonautologous graft material was excluded because the full text was only available in Chinese (abstract in English).[26]

### Studies included and characteristics

The three studies [Tables 1 and 2] compared pericranial autograft with synthetic allograft (GORE PRECLUDE MVP Dura Substitute (expanded PTFE)),[10] pericranial autograft with cadaveric dura,[1] and acellular human dura (AlloDerm (LifeCell Corp, Branchburg, NJ)) with bovine collagen matrix (Duragen, Integra Neuroscience, Plainsboro, NJ).[11] The studies were all published in peer-reviewed journals between 1997 and 2009 and were reported from single academic centers. Two of the articles were retrospective studies[10,11] and one employed a sequential, prospective analysis of the second cohort of 13 patients after switching from a nonautologous graft to an autologous graft after the first set of 13 patients.[1]

**Table 1:** Demographics of studies comparing two different types of dural substitutes in Chiari malformation decompressive surgery

Reference	Type of study	No. of patients	Grafts used (No.)		Mean age (range)		
		
			Group 1	Group 2	Group 1	Group 2	All
Attenello *et al*. 2009[10]	Retrospective, 10-year period not specified	67	Pericranium (40)	ePTFE (27)	10±4 yrs	12±5 yrs	11±5 yrs
Danish *et al*., 2006[11]	Retrospective, 2002-2004	101	Synthetic collagen (DuraGen) (56)	Acellular human dermis (AlloDerm) (45)	‐	‐	9 yrs (1-19)
Vanaclocha and Saiz-Sapena, 1997[1]	Sequential, prospective, 1989-1995	26	Pericranium (13)	Cadaveric Dura (13)	‐	‐	28.5 yrs (19-38)
Total		194	Pericranium (53)	Nonautologous (141)	‐	(1-38 years)	

yrs, years

**Table 2:** Sex and follow up in studies comparing two different types of dural substitutes in Chiari malformation decompressive surgery

Reference	Type of study	No. of patients	Sex (male)			Mean or median F/U (range)		
		
			Group 1	Group 2	All	Group 1	Group 2	All
Attenello *et al*. 2009[10]	Retrospective, 10-year period not specified	67	48%	26%	39%	16 mos. Median (6-28)	14 mos. Median (6-17)	16 mos. Median (6-24)
	Retrospective, 2002-2004	101	‐	‐	45%	‐	‐	10 mos. (1-24)
Danish *et al*., 2006[11]								
Vanaclocha and Saiz-Sapena, 1997[1]	Sequential, prospective, 1989-1995	26	‐	‐	53%	‐	‐	27 mos. (6-58)
Total		194	‐			(1-58 months)		

### Outcomes measured

The rates of superficial wound infections, bacterial meningitis, aseptic meningitis, CSF leaks, pseudomeningocele formation, and need for reoperation were evaluated. However, not all measures were reported in all three studies.

#### Study 1

In a report from the Johns Hopkins group, Attenello *et al*.[10] retrospectively compared the use of GORE PRECLUDE MVP dural substitute (expanded polytetrafluoroethylene [ePTFE] graft) with pericranium in 67 patients with a mean age of 11 years (standard deviation 5 years); 40 of these patients received an autologous pericranial graft. Variables of interest [Tables 3 and 4] included reoperation, wound infection, aseptic meningitis, pseudomeningocele, and CSF leakage. Indications for reoperation were not provided.

**Table 3:** Reoperation and infection rate of two different types of dural substitutes in Chiari malformation decompressive surgery

Reference	Reoperation			Aseptic meningitis			Wound infection		
			
	Group 1	Group 2	All	Group 1	Group 2	All	Group 1	Group 2	All
Attenello *et al*., 2009[10]	4/40 (10%)	0/27	4/67 (6%)	1/40 (3%)	1/27 (4%)	2/67 (3%)	0/40	0/27	0/67
Danish *et al*., 2006[11]	4/56 (7%)	2/45 (4%)	6/101 (6%)	‐	‐	‐	2/56 (4%)	1/45 (2%)	3/101 (3%)
Vanaclocha and Saiz-Sapena, 1997[1]	‐	‐	‐	0/13	2/13 (15%)	2/26 (8%)	‐	‐	‐
	Pericranium	Nonautologus grafts		Pericranium	Nonautologus grafts		Pericranium	Nonautologus grafts
Total	4/40 (10%)	6/128 (5%)		1/53 (2%)	3/40 (8%)		0/40	3/128 (2%)

**Table 4:** CSF-related complications associated with two different types of dural substitutes in Chiari malformation decompressive surgery

Reference	Pseudomeningocele			Incisional CSF leak		
		
	Group 1	Group 2	All	Group 1	Group 2	All
Attenello *et al*. 2009[10]	4/40 (10%)	6/27 (24%)	10/67 (15%)	2/40 (5%)	0/27	2/67 (3%)
Danish *et al*., 2006[11]	5/56 (9%)	5/45 (11%)	10/101 (10%)	1/56 (2%)	1/45 (2%)	2/101 (2%)
Vanaclocha and Saiz-Sapena, 1997[1]	0/13	6/13 (46%)	5/26 (20%)	0/13	2/13 (15%)	2/26 (19%)
Total	Pericranium 4/53 (8%)	Nonautologus grafts	Pericranium 2/53 (4%)	Nonautologus grafts
			22/141 (16%)			3/141 (2%)

No patient who received an ePTFE graft experienced a CSF leak or symptomatic pseudomeningocele; the rate of incisional CSF leaks was highest (but not significant) in the pericranium group. The rates of aseptic meningitis and wound infection were comparable between the two groups. Asymptomatic pseudomeningoceles were reported in 6 of 27 patients in the ePTFE group and in 4 of 40 patients in the pericranium group (22 vs. 10%, *P*=0.169). Four patients (10%) with a pericranium graft required reoperation versus none in the ePTFE group (*P*=0.090). A single surgeon performed all surgeries with ePTFE, and another surgeon performed all surgeries with pericranium.

This study reported notable findings related to resolution of syrinx and restoration of the physiologic dynamics of CSF flow. At a median follow up of 8 months, 100% of those with an ePTFE graft had physiologic hindbrain CSF flow patterns on cine magnetic resonance imaging (MRI) compared to 79% of those who had received autologous pericranium (*P*<0.05). A syrinx resolved in 80% of patients with ePTFE versus 52% of those with pericranium (*P*=0.140). The rate of symptom recurrence was highest for the pericranium group (27% vs. 11%, *P*=0.105).

#### Study 2

The second study[15] compared two types of allograft: DuraGen, a type I collagen matrix from bovine Achilles tendon, and AlloDerm, an acellular human dermis allograft. All patients treated with Alloderm (n=45) were treated before any patients were treated with a DuraGen onlay (n=56). No patient received both allografts. The study was retrospective and performed primarily in the pediatric population. The DuraGen was not sutured to the dura, but rather was placed on top of the dural defect. Despite this approach, the rates of clinically apparent pseudomeningoceles and incisional CSF leaks were lowest in the DuraGen group (not significant, Tables 3 and 4). Asymptomatic pseudomeningoceles were not routinely diagnosed because MRI was not performed routinely. The mean operative time was 37 min less in the DuraGen group (*P* <0.01). Rates of aseptic meningitis were not reported. Because harvesting pericranium can be difficult in children, the authors did not use it in this study although they stated that they considered it to be associated with the lowest risk of complications.

#### Study 3

This prospective study[19] was performed in the last 13 of 26 patients. The first 13 underwent Chiari decompression with freeze-dried cadaveric dura with a fibrin sealant (Tissucol). The authors converted to autologous pericranial grafts without sealant after the first 13 cases. The study was undertaken in the adult population (mean age 28.5 years, range 19-38 years). The investigators routinely performed MRI at follow-up visits. Their overall results favored the pericranial graft. Rates of pseudomeningocele formation were 46% and 0% in the cadaveric dura and pericranial graft groups, respectively (P=0.015). Two cases of aseptic meningitis and two cases of perioperative incisional CSF leaks were reported with cadaveric grafts. No complications were associated with the pericranial grafts. Rates of infection and reoperation were not reported. Positional headaches occurred for both groups but lasted 3.3 ± 1.7 days in those with pericranium and 17.3 ± 5.4 days in those with cadaveric dura (*P*<0.01).

Aggregate clinical outcomes for the three studies described are shown in Tables 3 and 4. The overall rates of reoperation (10% vs. 5%, *P*=0.25) and incisional CSF leaks (4% vs. 2%, *P*=0.61) tended to be higher in the pericranium group than in the nonautologous graft group. The overall rates of aseptic meningitis (8% vs. 2%, *P*=0.31), pseudomeningocele formation (16% vs. 8%, *P*=0.16), and wound infection (2% vs 0%, *P*=1.00) tended to be higher in the nonautologous graft group.

### Case example

#### History

**Figure 1:**
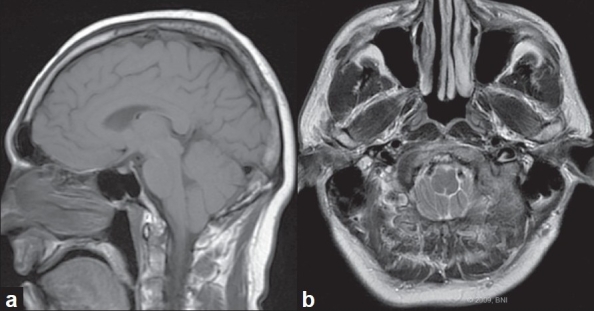
Preoperative (a) sagittal and (b) axial magnetic resonance images showing tonsillar herniation below the foramen magnum. [Used with permission from Barrown Neurological Institute]

A 41-year-old man presented with multiple complaints suggestive of a Chiari malformation. His most bothersome complaint was a 2-month history of increasing suboccipital headaches that worsened when he bent over. He reported radiating neck pain, intermittent numbness in his distal arms, increasing difficulty grasping objects and coordinating his finger manipulations, and intermittent dysphagia and dysarthria. On examination, he had hyperreflexia in his lower extremities and a unilateral Hoffman's sign. His sensation perception was decreased equally over his distal upper extremities in a nondermatomal pattern. MRI of his brain [Figure 1] showed a tonsillar herniation to the midportion of the C1 ring. The imaging of his brain and cervical spine revealed no other significant pathology.

#### Surgical technique

**Figure 2:**
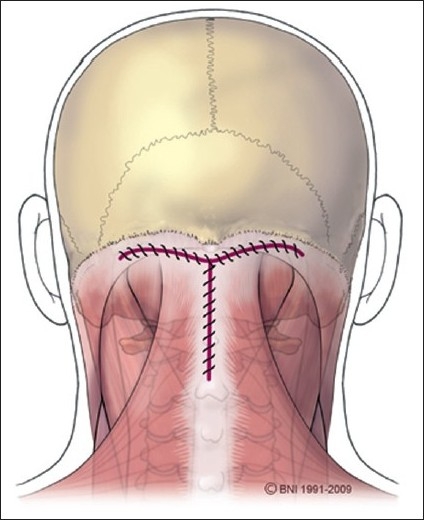
Schematic of "T" shaped fascial incision. [Used with permission from Barrown Neurological Institute]

The patient underwent a suboccipital craniectomy and laminectomy of C1. The midline incision spanned from approximately 2 cm cephalad of the superior aspect of the inion caudally to the spinous process of C2. Care was taken not to disrupt the underlying fascia of the occipital musculature or pericranium. The overlying tissues were undermined to allow a transverse incision to be made approximately 1 cm below the superior nuchal line. This strategy left a cuff of fascia and muscle for later use to recreate the muscular tension band and to allow a watertight closure of the fascia. A fascial incision starting at the midpoint of this transverse line was carried caudal to the superior aspect of C2, forming a T [Figure 2]. In this way, the inion is never visualized, only felt. Attention was then shifted to the pericranium above the inion.

**Figure 3:**
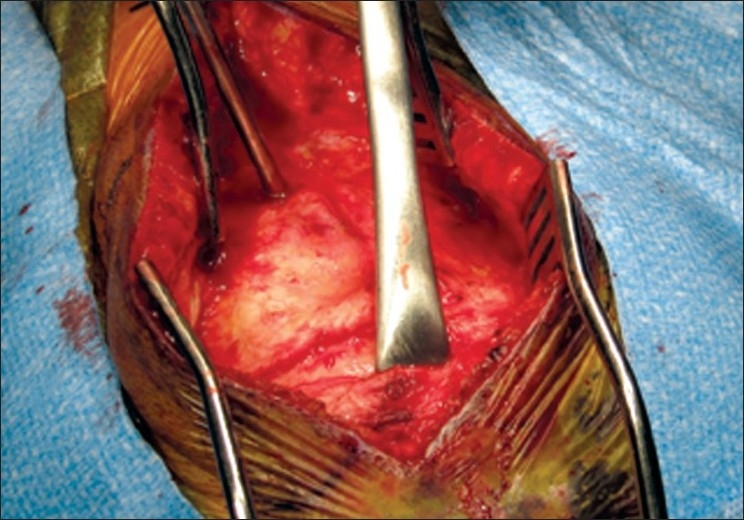
Intraoperative photograph of pericranial harvest. [Used with permission from Barrown Neurological Institute]

Using a handheld retractor/rake, the occipital scalp was undermined. A scalpel and electrocautery were used on "cut" to create the triangular graft (approximately 2.5 cm in height with a 3-cm base). A cautery tip bent at a 45° angle was utilized to maximize the graft size under the retracted scalp. Care was taken to keep the base of the triangular graft just above the superior nuchal line (approximate 0.5 cm above). The graft was freed from the cranium using a periosteal elevator tool with care being taken not to "buttonhole" it [Figure 3]. The graft was then kept in antibiotic-instilled saline.

**Figure 4:**
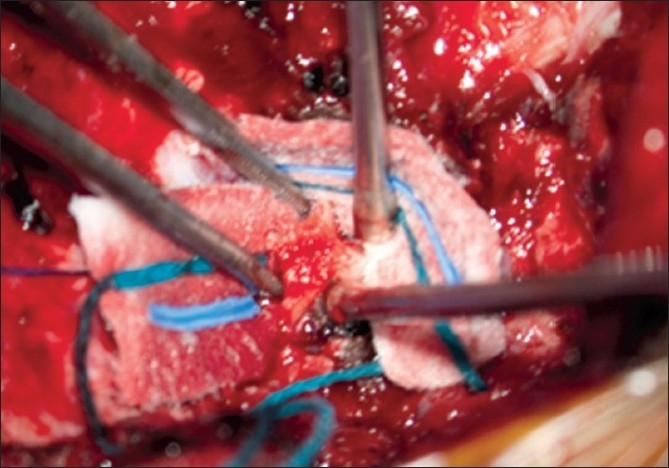
Intraoperative photograph of removal of lateral wall of foramen magnum. [Used with permission from Barrown Neurological Institute]

**Figure 5:**
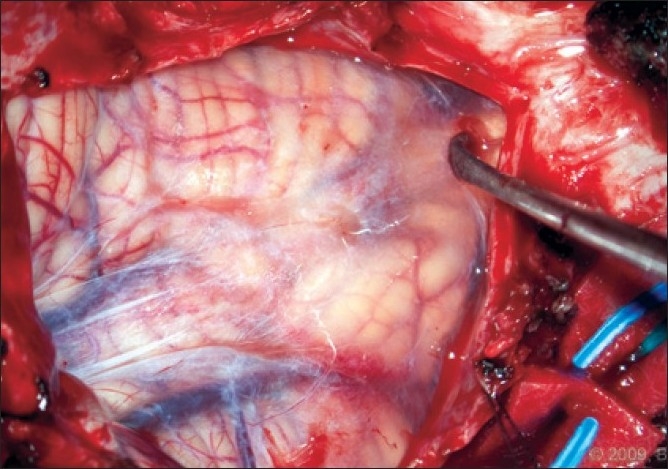
Intraoperative photograph of tonsils visualized through an intact arachnoid membrane. No adhesions are present and the caudal aspect of the tonsils can be seen. [Used with permission from Barrown Neurological Institute]

**Figure 6:**
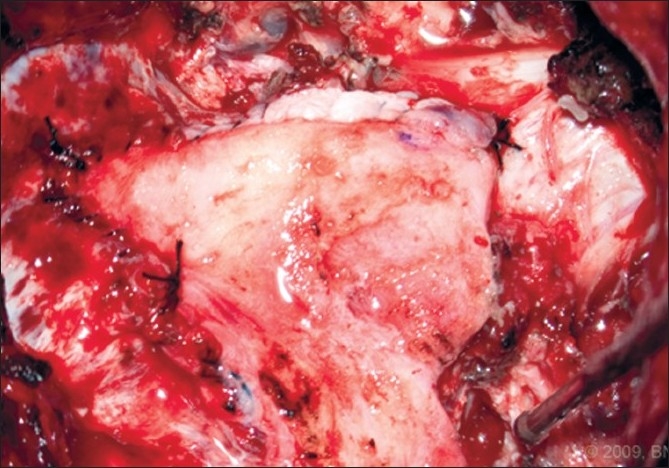
Intraoperative photograph of the pericranial graft sutured in place at the apices of the dural opening. [Used with permission from Barrown Neurological Institute]

After the suboccipital bone and posterior C1 arch were cleared, a wide bony suboccipital decompression with a C1 laminectomy was undertaken [Figure 4]. This decompression extended to the posterior aspect of the occipital condyles. The dura was then incised in a Y fashion. The arachnoid was kept intact. The tonsils were visible though the arachnoid and no adhesions were visualized [Figure 5]. The graft was sewed in place with the pericranial side facing the arachnoid using a running 4-0 Nurolon. The graft was tacked at each apex to assure one side would not be shorted [Figure 6].

Watertight closure was tested with a Valsalva maneuver. The graft and exposed dural were coated with a single application of DuraSeal. The muscles were reapproximated, and the fascia was closed in running, watertight fashion.

#### Postoperative course

**Figure 7:**
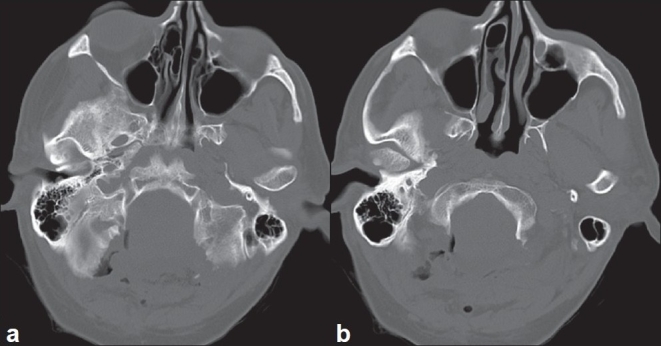
Postoperative axial computed tomography scans at slightly different cuts (a, b) showing the extent of bony decompression achieved. [Used with permission from Barrown Neurological Institute]

The patient's postoperative course was uneventful. Incisional pain resolved by the time of his follow-up visit. His preoperative head and neck pain resolved and his intermittent dysphagia and arm numbness resolved. He had no pseudomeningoceles, incisional problems, or infectious sequelae. His postoperative CT obtained soon after surgery showed that bony decompression was adequate [Figure 7]. We do not perform postoperative MRI unless clinically indicated.

## DISCUSSION

Although definitive data regarding the superior dural graft choice are lacking, this review shows some clinically relevant trends [Tables 3 and 4]. When available for harvest, autologous pericranium is associated with better rates of aseptic meningitis, wound infections, and pseudomeningocele formation compared to allografts. In contrast, allogenic and synthetic dural substitutes are advantageous when rates of reoperation and incisional CSF leakage are compared. Attenello *et al*.[10] demonstrated superior performance of ePTFE versus pericranium with respect to symptom resolution, syrinx resolution, and restoration of physiologic CSF flow at the foramen magnum. They contend that these findings can be linked to the microscopically smooth barrier of the ePTFE that minimizes neural attachments while maintaining an outer surface textured for biologic fixation.[10] However, the inherent difficulty of harvesting thin pediatric pericranium may have influenced their results. No aggregate clinically relevant differences reached statistical significance, and the limitations of this analysis are noteworthy. Nonetheless, advantages and drawbacks of both graft groups are readily apparent.

Autologous graft material has many theoretical advantages. In practice, the defects created by the securing suture are less likely to stretch or tear with use of pericranium compared to nonautologous material. Furthermore, unlike cadaveric dura or collagen, pericranium is living tissue, which enhances its function as a barrier. It more readily forms a watertight interface with dura when used with a running suture compared to synthetic materials, which are neither transformed nor vitalized.[1] This property should decrease rates of both pseudomeningocele formation and CSF leaks. The latter, however, also heavily depends on adequate fascial closure. These advantages of autograft are also supported by studies comparing harvested pericranium and synthetic substitutes for dural reconstruction after tumor resection.[25]

Despite these benefits, autologous grafts can place patients at risk for additional morbidity. Harvesting a pericranial graft involves a variable degree of additional wound extension that increases risks of wound breakdown, infection, local pain syndromes, and cosmetic complications. Although not reviewed here, fascia lata grafts require an additional operative site. Harvesting nuchal ligament for grafts can weaken support in the posterior neck.

The disadvantages of nonautologous dural grafts include an increased risk of hemorrhage, often with silastic grafts,[14,24,27] bacterial and viral transmission; Creutzfeldt-Jakob disease transmission, exclusively with cadaveric graft;[14,27‐36] eosinophilic-aseptic meningitis;[37] foreign body reaction and scarring;[14] increased wound healing time;[14] premature graft dissolution;[14] and wound dehiscence.[14] In the case of xenogenic pericardium, specifically bovine pericardium, the potential for bovine spongiform encephalopathy exists.[17] It has also been suggested that recurrence of symptoms after decompression and duraplasty with nonautologous graft may be related to postoperative fibrosis, adhesion formation, and subsequent recurrent hindbrain compression initiated by the graft itself.[27] We noted a higher aggregate rate of symptom recurrence in the allograft group in the three studies analyzed here, although data collection in this regard was intermittent.

Some nonautologus grafts may perform better than others. Rates of aseptic meningitis and wound infection likely depend on the interaction between the specific graft type and the patient's immune system. A recent study by Bejjani and Zabramski[21] , however did not support this assertion. In a camparison of porcine intestinal submucosa (Durasis, Cook Biotech Inc.) to historical data on various dural substitutes, they noted similar rates of CSF leak (~1.7%), wound infection (~3.4%), bacterial meningitis (~0%), and pseudomeningocele formation (~0%) in all subgroups.

Both the search methodology and aggregate analysis of this critical review were limited. Inclusion and exclusion criteria affect search results via both selection bias and inadvertent omission of studies that may have been relevant to the analysis. Furthermore, not all studies included data for all outcomes evaluated, and outcome definitions/indications (e.g., reoperation, wound infection, meningitis) were subjective, nonspecific, and variable across the study centers and providers. Differential timing of postoperative MRI creates biases in the detection of asymptomatic pseudomeningoceles. With respect to generalizability, the study results are limited to Chiari malformation patients who present to academic medical centers. Extrapolation of the effect of grafts on outcome measures for supratentorial surgery or Chiari decompression in a community population is limited.

### Additional study

Although it did not specifically investigate Chairi decompression, a study that compared to use of pericranial grafts and a synthetic subtitute in 124 patients who underwent both supra- and infratentorial brain tumor resection[25] provides additional data on rates of CSF leak and infection. In this study, internal CSF leaks (i.e. pseudomeningoceles) were significantly more frequent in the Neuro-Patch nonautologous group (n = 61) than in the pericranium group (n = 63) (13% vs. 1.6%, *P* < 0.05). The rate of deep wound infections (abscess, meningitis, osteitis, or empyema) was 15% in the Neuro-Patch group versus 5% in the pericranium group (*P* = 0.06). Furthermore, there was a strong relationship between internal CSF leakage and infection. Of the eight patients in the Neuro-Patch group with an internal CSF leak, four became infected. Of the nine patients with a deep wound infection in the same group, four had internal CSF leaks. The evidence is suggestive that nonautologus grafts are more likely to cause CSF complications and infection. Nonetheless, we do not find the results of studies investigating dural substitutes for supratentorial pathology to be generalizable to Chiari surgery.

### Institutional preference

At our institution, all surgeons who perform Chiari malformation surgery employ bony decompression and the use of duraplasty. We favor a wider extent of bony decompression and often remove the bone at the medial aspect of the posterior occipital condyle to allow maximal decompression of the lateral foramen magnum.[18] Confirmation of adequate lateral decompression is obtained intraoperatively by carefully palpating the lateral foramen magnum with a No. 4 Penfield dissector to ensure that the remaining bone is flush with the occipital condyle.

We find that duraplasty is essential for successful Chiari surgery because it creates an artificial cisterna magna where one was not previously present.[1] Opening of the arachnoid varies by surgeon. Tonsillar resection/shrinkage is seldom performed, and the choice of graft is distributed evenly between autograft and allograft (synthetic or xenogenic). For the senior author (VKHS) pericranium duraplasty is used in conjunction with subarachnoid dissection of arachnoid veils. We concur with Stevens *et al*.[14] that pericranium is "generally nonimmunogenic, nontoxic, rapidly integrated into native tissues, flexible, strong, easily suturable, and inexpensive." Great care is taken to avoid allowing hemorrhage from superficial tissues to enter the subarachnoid space.

In the case example presented here, a duraplasty was employed without arachnoid opening, because decompression was adequate inferior to the caudalmost aspect of the tonsillar herniation [Figure 4]. This technique provides the advantage of tonsillar decompression without allowing communication between the subarachnoid space and outside compartments. As an additional option employed by a minority of surgeons at our institution, a dissected arachnoid can be resutured to prevent CSF-related complications. We do not routinely use ultrasonography to evaluate the adequacy of decompression of the tonsils and normalization of CSF flow dynamics.

Authors have advocated the use of electrocautery in procuring pericranial grafts.[14] We believe that a scalpel is an acceptable alternative. We agree with Stevens *et al*. that pericranium can be harvested without extending the incision significantly above the inion and superior nuchal line, thus avoiding injury to the occipital arteries and nerves.[14] The use of a hand-held retractor to develop a subgaleal plane with Metzenbaum scissors beneath the rostral limits of the incision facilitates this dissection. We harvest a graft that is approximately 2.5 cm × 2.5 cm in dimension, perform the harvest before the bony decompression or dural opening, and soak the graft in antibiotic-saline irrigation. We also find that a T incision of the fascia based below the superior nuchal line allows for more robust watertight closure of the fascia to contain possible pseudomeningocele formation. This fascia incision also creates an anchor point to which the occipital musculature can be fixed, preventing loss of the occipital-to-C2 tension band.

## RECOMMENDATIONS AND CONCLUSIONS

No class I or II level evidence addresses the extent of bony removal, the need for a dural opening, or the extent of intradural manipulation required in Chiari malformation surgery. Some of the available Class III data, however, can begin to guide decision making. Despite considerable variability in surgeons' practice patterns, an increasing number of investigations and community neurosurgeons advocate the use of duraplasty in Chiari decompression. The reduction in reoperation rates must be weighed against the risk of CSF-related complications. In our opinion, the creation of an artificial cisterna magna via duraplasty is imperative. Opening the arachnoid with or without reapproximation is not well studied and is left to the discretion of the operating surgeon.

When duraplasty is employed, the literature does not strongly support the superiority of either autologous or nonautologous grafts. This lack of consensus was recapitulated in the three series analyzed here. Nonetheless, we believe that when the pericranium is available and of good quality, it should be utilized. It is non-immunogenic, inexpensive, and capable of creating a watertight closure with the dura. The quality of a pericranial graft is a relevant concern. While Vanaclocha *et al*.[19] demonstrated significant advantages to the use of pericranial graft in adults, the superiority of ePTFE was demonstrated in the larger of the two pediatric comparison studies.[4] These discrepancies are likely attributable to increases in the quality and thickness of the pericranium with maturity. Future randomized studies with large numbers and the power to resolve differences in the complication rates of meningitis, wound infections, and reoperations (which are all often less than 5%) are warranted to establish the superiority of pericranium over other available dural substitutes.
